# Neural Correlates of Self-Appraisals in the Near and Distant Future: An Event-Related Potential Study

**DOI:** 10.1371/journal.pone.0084332

**Published:** 2013-12-20

**Authors:** Yangmei Luo, Todd Jackson, Xiaogang Wang, Xiting Huang

**Affiliations:** 1 Key Laboratory of Cognition and Personality (SWU), China Ministry of Education, Chongqing, China; 2 Faculty of Psychology, Southwest University, Chongqing, China; University of Bologna, Italy

## Abstract

To investigate perceptual and neural correlates of future self-appraisals as a function of temporal distance, event-related potentials (ERPs) were recorded while participants (11 women, eight men) made judgments about the applicability of trait adjectives to their near future selves (i.e., one month from now) and their distant future selves (i.e., three years from now). Behavioral results indicated people used fewer positive adjectives, more negative adjectives, recalled more specific events coming to mind and felt more psychologically connected to the near future self than the distant future self. Electrophysiological results demonstrated that negative trait adjectives elicited more positive ERP deflections than did positive trait adjectives in the interval between 550 and 800 ms (late positive component) within the near future self condition. However, within the same interval, there were no significant differences between negative and positive traits adjectives in the distant future self condition. The results suggest that negative emotional processing in future self-appraisals is modulated by temporal distance, consistent with predictions of construal level theory.

## Introduction

Mental time travel is one of the most fascinating characteristics of the human mind [1]–[3]. Mental time travel results in temporal selves; that is, people construct their self-identity by recalling their past and imagining a hypothetical future self [4], [5]. Moreover, perceptions of temporal selves can enhance self-regulation abilities and activate goal-related behaviors. For example, simulations of hypothetical future selves helped middle school students increase their school involvements and adaptive behaviors [6]. Perceptions of temporal selves can also influence decisions. For instance, people who perceive and treat the future self differently from the present self are prone to making short-sighted decisions [7]. Therefore, it is important to understand how people construct and represent their temporal selves. In this study, we focused on people's perception of the future self.

There is considerable evidence that people's future self is dominated by favorable self-views. People believe that they are more likely to experience positive events and less likely to experience negative events compared to their peers [8], [9]. Positive future events are also generated more easily and quickly than negative future events [10] and people evaluate their future selves as having more desirable traits than their present and past selves [11]. Notably, the dominance of favorable self-views for the future self has important implications for mental health. Specifically, perceptions of a moderately desirable future may promote motivation, mental health and well-being [12], [13]. In contrast, a pessimistic view to the future is related to depressive disorders [14], [15].

Temporal distance influences perceptions of the future self. Although people view the future self favorably, the degree of favorability is affected by temporal distance. Construal level theory has been employed to explain mechanisms underlying the effect of temporal distance on future self-appraisals [16]. From this perspective, whereas the near future self is associated with a low-level, concrete construal, the distant future self is associated with a high-level, abstract construal [17]–[19]. To elaborate, the near future self-construal is more grounded in concrete events and comprises complex representations of the self that include both positive and negative self-construals. In contrast, because the distant future self is relatively remote from direct life experiences and people have a tendency to view the future in a positive light [8], [10], [20], [21], distant future self-views should be even more favorable and positive. In support of construal level theory, Heller et al. (2011) found perceptions of (1) affect, (2) traits, and (3) narratives of one's distant future self (i.e., three years from now) were more positive and less variable than perceptions of one's near future self (i.e., a month from now) in three independent studies [16]. Similarly, Kanten and Teigen (2008) found that people predicted having a more favorable future self in two years' as opposed to six months' time [11].

However, research on perceptions of near versus distant future selves is not uniformly consistent. Theorists using temporal self-appraisal theory have also identified circumstances in which people might expect better outcomes for the distant future self and note remote future selves are unlikely to be disparaged [4]. At the same time, however, people “may simply be motivated to exaggerate the glories of psychologically proximal future selves more than those of distant future selves” (Peetz & Wilson, 2008: 2097) [4], to maintain favorable current self-regard [4], [22], [23]. In support of these ideas, Wilson et al. (2012) manipulated subjective temporal distance of a future self by holding objective or calendar time constant, and found participants predicted having more favorable personal attributes at a close future time than a distant future time [23].

In light of mixed findings, further research is warranted to evaluate the extent to which attributes of the near or distant future self are perceived as more favorable. Furthermore, much of the associated research has focused on perceived behavioral differences between near and distant future self-appraisal [11], [16], [23], so it is less clear whether self-appraisals of near and remote future distant selves correspond to distinct underlying neural responses. Evaluation of neural correlates related to evaluating attributes of near and distant future selves can elucidate how temporal distance is related stage of processing self-appraisals, because such judgments may occur within a few hundred milliseconds, well before behavioral responses. With high time resolution on the order of milliseconds, ERPs provide an excellent means to evaluate the time course of future self-appraisal processing as a function of temporal distance.

In relation to components of ERP, the late positive component (LPC) occurs between 300 and 1200 ms after stimulus onset [24], and is theorized to indicate emotional processing. The amplitude of LPC is usually larger for both positive and negative than for neutral stimuli [25], [26]. However, for direct comparisons of LPC amplitudes elicited by positive and negative stimuli, results have been mixed. Some studies have found negative stimuli elicit larger LPC [27]–[29] than positive stimuli do, while the reverse pattern [30], [31] or null effects [25] have also been observed. Despite such discrepancies, the positivity of LPC to emotional stimuli is associated with later controlled cognitive evaluation of emotional meanings of stimuli [26], [29], [32], [33]. Furthermore, LPC effects have been implicated in episodic memory retrieval and mental imagery [34]–[36]. For example, Rugg et al. (1998) found LPC amplitude was larger for more deeply encoded items [34]. Also, Kanske & Kotz (2007) found that concrete words, which are prone to mental imagery, evoked an LPC effect while there was no effect on abstract words [37]. In another relevant study, negative words elicited a larger positive LPC deflection relative to positive words when evaluating present and past selves. However, in the same interval, there was no a significant LPC effect when evaluating the future selves. It was speculated that the negative words could evoke more specific episodic events in the past and present selves relative to future selves [28].

Drawing upon the methodology and hypotheses from Heller et al.'s [17] research as well as contentions of construal level theory that the near future self-construal is relatively concrete and comprised of complex representations that include both positive and negative self-conceptions while the distant future self is more abstract and idealized in a more uniformly positive way [16]–[19], we assessed interactions between temporal distance and valence on behavioral and electrophysiological data. Specifically, it was hypothesized that people would have more favorable distant future self-view than near future self-view. Moreover, negative adjectives for the near future self should elicit a larger amplitude of LPC than positive adjectives, while emotional adjective should have no effect within the distant future self condition. To assess these hypotheses, participants evaluated their near and distant future selves across a series of positive and negative trait adjectives within an ERPs experiment. Following Heller et al., [16], the near future self were operationalized as the self in one month's time, and the distant future self was operationalized as the self in three years' time.

## Materials and Methods

### Ethics Statement

The School of Psychology Research Ethics Committee of Southwest University (SWU) granted ethical approval for the study and all participants gave written informed consent to the experimental procedure. They were informed that they had the right to withdraw at any time during the experiment.

### Participants

ERP recordings were obtained from 19 undergraduates (eleven women, eight men) aged 19–22 years (mean age, 21.01 years) from SWU in Chongqing, China. ERP data from one participant was removed due to excessive artifacts. All participants engaged in the experiment as paid volunteers, had no history of current or past neurological or psychiatric illness, and had normal or corrected-to-normal vision.

### Stimuli

The 40 positive and 40 negative trait adjectives used in the current study were the same as those used in previous research [27]. Mean valences were 5.61 (*SE* = 0.04) for positive traits adjectives and 2.72 (*SE* = 0.07), for negative traits adjectives using a 7-point rating scale anchored at 1 (least desirable) and 7 (most desirable). Positive and negative trait adjectives were matched for familiarity, meaningfulness, and complexity (i.e., number of strokes needed to write the Chinese character).

### Procedure

Participants made self-judgments on the same set of adjectives in two conditions. Specifically, they were instructed to decide whether or not the 80 adjectives described characteristics they might have one month from now (Near Future Self), and characteristics they might have three years from now (Distant Future Self). Each adjective was presented twice within the two self conditions (i.e., 160 trials per condition). Within each condition, there were four blocks of 40 trials each. The interval between trials was 1000–1500 ms. Conditions and blocks were presented in random order.

To prime the corresponding temporal self, participants were asked to describe in writing their image of the corresponding temporal self for two minutes before initiating each condition [5]. Subsequently, all trials for that self-judgment condition followed the priming task. First, a fixation point appeared for 500–750 ms in the center of the screen and was followed by a cue for the temporal self condition presented for 250 ms (“the self one month later” “the self three years from now”). After a 400–800 ms interval, a trait adjective was presented for 3000 ms. Participants were asked to respond as accurately and quickly as possible regarding the extent to which each adjective described their near (or distant) future self on a 4-point rating scale (very unsuitable = 1, unsuitable = 2, suitable = 3, very suitable = 4). Based on Moran et al. [38], responses of 1 or 2 were considered low in self-relevance and responses of 3 or 4 were considered high in self-relevance.

After the ERP procedure, more information was collected on participant performance during the task. Specifically, participants were asked to rate the frequency with which specific events came to their mind when evaluating their near and distant future selves (using a 7-point rating scale: 1 =  not at all, 7 =  very frequently). In addition, participants completed a modified version of the Future Self-Continuity Scale [39]. This scale assessed the connectivity between the current self and each type of future self with depictions of two circles that ranged from no overlap to almost complete overlap. Participants selected the circle pair that best described how connected they felt to a future self one month later or three years from now.

### Event-related potential recording and analysis

Continuous brain electrical activity was recorded from 64 scalp sites using tin electrodes mounted in an elastic cap (Brain Product, Munchen, Germany), with the reference on left and right mastoids. The vertical electrooculogram (EOG) was recorded with electrodes placed above and below the left eye. All inter-electrode impedance was maintained below 5 kΩ. The electroencephalogram (EEG) and EOG were amplified using a DC-100Hz bandpass and continuously sampled at 500 Hz/channel for off-line analysis. Eye movement artifacts were corrected with the Gratton–Coles algorithm using the EOG data [23]. After this, trials with EOG artifacts (mean EOG voltage exceeding ±100 µV) and those contaminated with artifacts due to amplifier clipping, bursts of electromyographic (EMG) activity, or peak-to-peak deflection exceeding ±100 µV were excluded from averaging. Following from other published studies [40], [41], a 16 Hz low pass filter was used.

Respective EEG averages for the four conditions of interest (positive-near future self, negative-near future self, positive-distant future self, and negative distant future self) were calculated. The averaged epoch for ERPs was 1200 ms including a 200 ms pre-response baseline. Electrodes of interest were analyzed with a repeated-measures analysis of variance (ANOVA) (2: Near, Distant x 2: Positive, Negative x 9: Fcz, Fc3, Fc4, Cz, C3, C4, CPz, CP3, CP4). When a main effect was found, a Bonferroni-corrected post-hoc t-test for multiple comparisons was used to determine the significance of the difference for each pair-wise comparison. For all analyses, *p* values were corrected for deviations according to the Greenhouse–Geisser method.

## Results

### Behavioral performance

Judgments of each future self condition were collapsed into high (3 and 4 responses) and low (1 and 2 responses) self-relevance categories. Mean proportions of “high” responses given for positive and negative traits in each judgment condition are shown in Table 1. A repeated-measures ANOVA (2: near, distant x 2: positive, negative) found a main effect of valence (*F* (1, 18) = 319.75, *p*<.001, 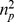 = .95) that was qualified by an interaction between temporal distance and valence (*F* (1, 18) = 12.30, *p* = .003, 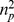 = .41). No significant main effect of temporal distance was found (*F* (1, 18) = .09, *p* = .925, 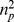 = .00). A simple effects analysis of temporal distance x valence interaction showed that “high” responses for positive trait adjectives in the near future self condition (*M* = 87.83%, *SE* = 2.67%) were significantly lower than those in the distant future self condition (*M* = 92.43%, *SE* = 1.85%) (*t*(18) = −2.64, *p* = .017, Cohen's *d* = −.46). Negative trait adjectives showed the opposite pattern: “high” responses in the near future self condition (*M* = 14.67%, *SE* = 3.38%) were significantly higher than in the distant future self condition (*M* = 10.26%, *SE* = 2.86%) (*t*(18) = 2.85, *p* = .011, Cohen's *d* = .32).

**Table 1:** Behavioral measures as a function of future self condition.

	Near future self	Distant future self
Proportion of “high” responses (%)		
Positive traits	87.83(2.67)	92.43(1.85)
Negative traits	14.67 (3.38)	10.26(2.86)
Response times (ms)		
Positive traits	917.97(42.58)	907.98(40.34)
Negative traits	953.46(39.72)	976.28(45.74)
Connectivity with future self	5.79(.36)	4.47(.29)
Frequency of specific events	5.05(.30)	4.16(.34)

*Note*: standard errors are shown in parentheses

A repeated-measures ANOVA (2: near, distant x 2: positive, negative) on reaction times found a main effect for valence (*F* (1, 18) = 16.23, *p*<.001, 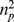 = .47) (Table 1). RTs for positive traits (*M* = 890.12, *SE* = 39.00) were significantly faster than RTs for negative traits (*M* = 940.36, *SE* = 41.39) (*t*(18) = −4.03, *p* = .001, Cohen's *d* = −.29). However, the main effect for time (*F* (1, 18) = .78, *p* = .387, 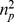 = .04) and temporal distance x valence interaction (*F* (1, 18) = 3.01, *p* = .100, 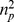 = .14) were not significant.

On self-report measures assessed following the ERPs session, paired samples t-tests showed that participants evaluated their present selves as more connected with their near future selves (*M* = 5.79, *SE* = .36) than their distant future selves (*M* = 4.47, *SE* = .29) (*t*(18) = 3.51, *p* = .003, Cohen's *d* = .93). Finally, as expected, specific events were more likely to come to mind when evaluating the near future self (*M* = 5.05, *SE* = .30) than the distant future self (*M* = 4.16, *SE* = .34) (*t*(18) = 2.30, *p* = .034, Cohen's *d* = .63).

### Event-related brain potential waveforms analysis

**Figure 1:**
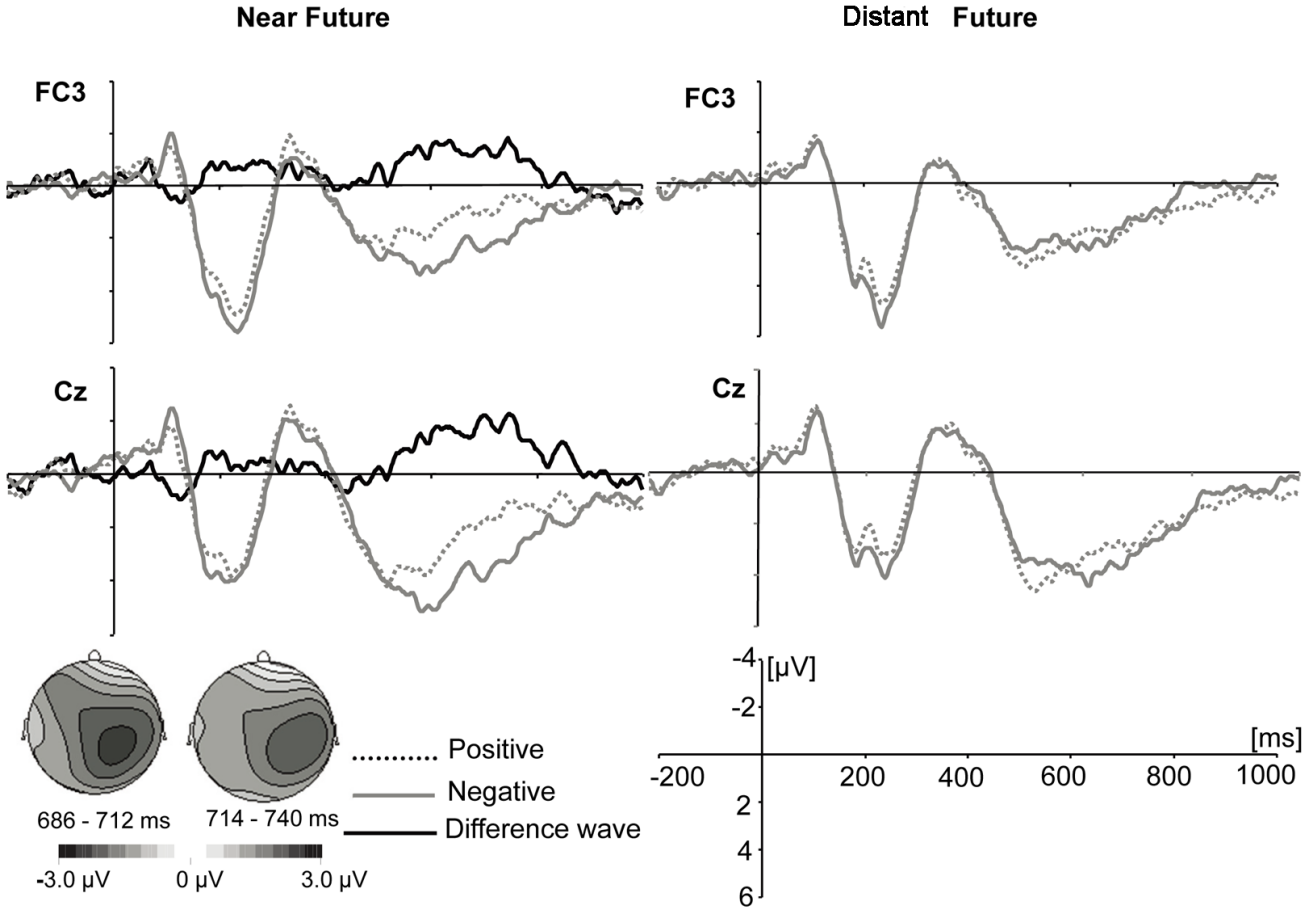
Grand average event-related brain potentials and topographical maps for the difference wave. A: Grand average event-related brain potentials. ERPs evoked by positive and negative traits for the near and distant future condition, and the difference wave (negative near future condition-positive near future condition) at FC3 and Cz. B: topographical maps of the voltage amplitudes for the difference wave at 686–712 and 714–740 ms.

As shown in Figure 1, the N1 (50–150 ms), P2 (150–300 ms), N2 (300–400 ms) and late positive component (LPC) were elicited by both near and distant future self conditions.

Main effects for trait valence and temporal distance were not significant for N1, P2 or N2. From ERP waveforms, we found that, relative to positive traits, negative traits for the near future self elicited a more positive ERP deflection than for distant future selves in the interval between 550 ms and 800 ms(LPC). A repeated-measures ANOVA (2: near, distant x 2: positive, negative x 9: electrode site) predicting the amplitude of LPC found a marginal effect for valence (*F* (1, 17) = 4.25, *p* = .055, 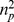 = .20) and a significant temporal distance x trait valence interaction (*F* (1, 17) = 5.20, *p* = .036, 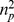 = .23). None of the other comparisons were significant (all *p*s>.05). Mean LPC amplitudes elicited by negative trait adjectives (*M* = 3.22, *SE* = 1.31) were more positive than those elicited by positive trait adjectives (*M* = 1.66, *SE* = .99) when evaluating the near future self (*t*(17) = 2.78, *p* = .013, Cohen's *d* = .32). There was no such difference between negative traits (*M* = 2.85, *SE* = 1.02) and positive traits (*M* = 2.40, *SE* = .87) in the distant future self condition (*t*(17) = .85, *p* = .406, Cohen's *d* = .11). As shown in Figure 1, these differences were evident in the central-parietal cortex. Neither main effects nor interactions for mean LPC amplitudes were significant between 400–550 ms and 800–1000 ms.

Pearson correlations were run to explore whether LPC amplitudes (550 ms and 800 ms) correlated with reported psychological connectivity and event recall frequency when participants evaluated their future selves. No significant correlations were found (see Table 2), but the highest correlation for ERP amplitude was for frequency of specific events recalled in the near negative future self condition (*r* = .317, *p* = 0.2) relative to other conditions.

**Table 2:** Correlations between LPC amplitude and connectivity and event recall frequency in near and distant future self conditions.

The LPC amplitude		Connectivity		Frequency	
		Near Future	Distant Future	Near Future	Distant Future
Near Positive Future	*r*	.039	.026	.251	.07
	*p*	.878	.917	.315	.784
Near Negative Future	*r*	.063	−.027	.317	.215
	*p*	.804	.915	.2	.392
Distant Positive Future	*r*	.022	−.133	.095	−.124
	*p*	.932	.598	.709	.623
Distant Negative Future	*r*	.209	.086	−.042	−.094
	*p*	.404	.736	.867	.711

## Discussion

The current study examined both behavioral and neural responses related to processing of future self-appraisals as a function of temporal distance. Behavioral results showed people used fewer positive adjectives and more negative adjectives to describe their near future selves (i.e. one month from now) relative to their distant future selves (i.e. three years from now). Moreover, while making judgments, participants reported specific events came to mind more often and they felt more psychologically connected to the near future self rather than the distant future self. Behavioral results about future personality attributes were consistent with select previous studies indicating people have more favorable distant future self-appraisals than near future self-appraisals [11], [16]. These findings demonstrated how people's predictions of their future personality might depend on how far they project into the future, with a more highly optimistic bias regarding the distant future self, at least within this experimental paradigm.

Electrophysiological results indicated ERP components (i.e., N1, P2 and N2) associated with early stages of visual and semantic processing did not differ between near and distant future self conditions. However, in the current study, temporal distance significantly interacted with emotional valence to predict LPC. For the near future self, larger LPC amplitudes were elicited by negative trait adjectives relative to positive trait adjectives from 550 ms to 800 ms after stimulus onset over the central-parietal region. In contrast, the distant future self did not show a significant difference in LPC elicited by negative versus positive traits within this interval.

In emotional electrophysiological studies, it is well established that the LPC reflects the elaborate and controlled late processing of emotional stimuli. In this stage, information is represented and analyzed more fully because more details including past or recent episodic experiences are referenced [27], [29], [32]. Also, the LPC effect has been linked to episodic memory retrieval and mental imagery [28], [34]–[36]. For example, West, & Holcomb (2000) found an LPC effect was most evident between 550 and 800 ms in an imagery task relative to semantic decision and surface characteristics tasks [36]. Based on tenets of construal level theory that near future self-representations are more complex and comprised of both positive and negative attributes while distant future self-representations are more idealized and uniformly positive, it is possible that negative traits in the near future self condition evoked more specific episodic thoughts and imagery relative to negative traits evoked in the distant future self condition. This conjecture is consistent with behavioral results indicating participants recalled more specific events coming to mind and more perceived connectedness in the near future self condition compared to the context of a distant future.

In sum, our behavioral and electrophysiological results were consistent with central assumptions of construal level theory. From this perspective, the near future self is related to a complex, low level, concrete construal characterized by positive and negative attributes while the distant future self is related to a high level, abstract construal characterized by idealized, schematic thinking [17]. The near future self construal is relatively more grounded in mixed valence experiences of daily life compared with the more highly idealized distant future self construal. Findings that participants used relatively fewer positive adjectives and more negative adjectives, recalled more specific events and perceived relatively increased connectedness in the near future self condition were in line with assumptions of the construal approach.

Nonetheless, these results were also partially consistent with temporal self-appraisal theory [4], [22], [23] which posits people show optimistic biases towards *both* temporal selves due, in part, to motivation to protect current self-regard (self-enhancement) [4], [22]. In addition, the results revealed that people felt relatively more connected and recalled more specific episodic events in the near future than distant future self condition, a finding that bolsters Wilson et al. 's claim that the near future self is more important to the current self-regard than the distant future self [23].

Furthermore, electrophysiological results revealing an LPC effect in the near future self condition but not in the distant future self condition suggest negative traits adjectives evoke more episodic thoughts in near future self-appraisals relative to distant future self-appraisals, consistent with construal level theory. These findings may also support previous research showing how level of abstraction can interact with emotional valence. Specifically, Kanske & Kotz (2007) found that concrete negative words (low level construal) elicited larger LPC amplitude than concrete neutral or concrete positive words while there was no emotional effect on abstract words [37].

Although the results provided further evidence that temporal distance modulated future self-appraisals, it is worth noting limitations of the present study and specific directions for future work. First, effect sizes were relatively small, perhaps because people had relatively favorable future self-views in both future self conditions. On a related note, it is not clear how well findings generalize beyond the current methodology, given that other researchers have also found replicable effects of a more favorable near future self using different research measures and designs [23]. Future research is needed to clarify the robustness of effects across study paradigms including those that feature manipulations of subjective time rather than calendar time [23]. Third, following previous studies [33], it would be useful to separate self-referential processing from emotional valences of trait adjectives in future work. Fourth, based upon thoughtful suggestions from a reviewer, optimal experimental designs for future study should include present self-appraisals as a control condition that would permit the examination of temporal distance on self-appraisals in a more precise way. Finally, a deeper understanding of these processes may result from extensions to depressed or anxiety disordered participants, given that these groups tend to have relatively more pessimistic views of their future selves (e.g. [42]).

## Conclusions

In summary, the present study demonstrated that neural substrates of future self-appraisals were modulated by temporal distance. The results showed that, when reflecting on the near future self, negative trait adjectives elicited more positive ERP deflections than positive trait adjectives in the interval between 550 and 800 ms (LPC). Conversely, there were no significant differences in ERP deflections elicited by negative and positive traits adjectives when evaluating the distant future self in the same interval. The findings suggest temporal distance modulates negative emotional processing in future self-appraisals, which is predicted by construal level theory. Future research is needed to understand how these results generalize to changes in subjective time and populations that veer toward pessimistic future self-appraisals.
